# Burden of migraine with acute medication overuse or psychiatric comorbidities and treatment with CGRP pathway-targeted monoclonal antibodies: A review

**DOI:** 10.1097/MD.0000000000033874

**Published:** 2023-06-09

**Authors:** Christopher Rhyne, Joshua M. Cohen, Michael J. Seminerio, Karen Carr, Lynda J. Krasenbaum

**Affiliations:** a Diamond Headache Clinic, Chicago, IL; b Teva Branded Pharmaceutical Products R&D, Inc., West Chester, PA.

**Keywords:** acute medication overuse, calcitonin gene-related peptide, comorbidity, depression, migraine, monoclonal antibody, prevention

## Abstract

Migraine is a complex and often debilitating neurological disease that affects more than 1 billion people worldwide. It is characterized by moderate-to-intense, throbbing headache attacks that are worsened by activity and is associated with nausea, vomiting, and sensitivity to light and sound. Migraine, ranked the second leading cause of years lived with disability by the World Health Organization, can diminish patients’ quality of life and bring significant personal and economic burden. Furthermore, migraine patients with a history of acute medication overuse (AMO) or psychiatric comorbidities, such as depression or anxiety, may experience even greater impairment and burden, and their migraine may be more difficult-to-treat. Appropriate treatment of migraine is essential to reduce this burden and improve patient outcomes, especially for those with AMO or psychiatric comorbidities. There are several available preventive treatment options for migraine, though many of these are not migraine-specific and may have limited efficacy and/or poor tolerability. The calcitonin gene-related peptide pathway plays a key role in the pathophysiology of migraine, and monoclonal antibodies that target the calcitonin gene-related peptide pathway have been developed as specific preventive treatments for migraine. Four of these monoclonal antibodies have been approved for the preventive treatment of migraine after demonstrating favorable safety and efficacy profiles. These treatments offer substantial benefits for migraine patients, including those with AMO or common psychiatric comorbidities, by reducing monthly headache days and migraine days, days of acute medication use, and disability measures, as well as improving quality of life.

## 1. Introduction

### 1.1. Epidemiology and burden of migraine

Migraine, which is characterized by headache attacks associated with nausea, vomiting, and sensitivity to light or sound,^[1,2]^ can be categorized by the frequency of attacks as episodic migraine (EM; headache on <15 d/mo) or chronic migraine (CM; headache on ≥15 d/mo for >3 months, with ≥8 d/mo meeting the migraine criteria as defined by the International Classification of Headache Disorders-3^[3]^). Migraine is one of the 3 most burdensome neurological disorders in the United States, affecting approximately 68.5 million people and resulting in 2.4 million disability-adjusted life years.^[4]^ Further, according to the 2019 Global Burden of Disease, Injuries, and Risk Factors study, migraine was ranked the second leading cause of disability worldwide, resulting in 4.9% of total years lived with disability.^[5]^ By comparison, low back pain was the leading cause of disability globally, resulting in 7.6% of total years lived with disability. Similarly, average annual direct medical costs per patient for patients with migraine ($11,010) are higher compared with patients without migraine ($4436)^[6]^ and are generally higher compared with those for patients with other common chronic conditions (e.g., diabetes, $3219–$4674; chronic obstructive pulmonary disease, $3968–$6491; asthma, $989–$3069).^[7]^ Migraine is associated with a number of comorbidities, including depression and anxiety, which may be associated with an increase in the severity and burden of migraine.^[8,9]^ Similarly, patients who are overusing acute migraine medications (acute medication overuse [AMO]) due to challenges with managing their migraine symptoms may experience greater migraine severity and burden.^[10]^ Patients with migraine with AMO and psychiatric comorbidities may have greater costs and health care resource utilization.^[10–12]^

### 1.2. Pathophysiology of migraine

Migraine has been shown to be associated with increased activity and sensitivity of the trigeminovascular system.^[2]^ This hyperexcitability leads to the activation of nociceptors in the meningeal blood vessels, cerebral arteries, and sinuses, causing headaches and other migraine-related symptoms.^[2]^ Calcitonin gene-related peptide (CGRP) plays a crucial role in migraine pathogenesis.^[13]^ CGRP is expressed in the central and peripheral nervous system and may modulate the trigeminovascular system, leading to pain and other symptoms associated with migraine.^[13,14]^

This review aims to describe the impairment and burden experienced by patients with migraine with AMO or psychiatric comorbidities, as well as barriers to diagnosis. The use of CGRP pathway-targeted monoclonal antibodies for the preventive treatment of migraine, particularly migraine with AMO or depression or anxiety, will also be discussed.

## 2. Burden of psychiatric comorbidities and AMO in migraine

### 2.1. Comorbidities of migraine

Psychiatric diseases, including depression and anxiety, are common comorbidities associated with migraine.^[15,16]^ Breslau and colleagues showed that patients with major depression are >3 times as likely to have migraine as those without depression, and patients with migraine are nearly 6 times as likely to have major depression as those without migraine.^[17]^ Furthermore, the presence of comorbid anxiety and depression in patients with migraine is associated with increased migraine-related disability, reduced quality of life, and greater work productivity and activity impairment.^[18]^ Lipton and colleagues found that rates of moderate-to-severe migraine-related disability were higher among migraine patients with anxiety and/or depression (43–61%) compared to migraine patients without anxiety or depression (28%).^[16]^

### 2.2. Acute medication overuse

Overuse of acute medications for migraine (AMO), defined by use of simple analgesics or combination medications on ≥15 d/mo or use of triptans, ergots, opioids, or combination analgesics on ≥10 d/mo,^[19]^ is also associated with increased migraine severity, risk for progression from EM to CM, reduced quality of life, and an increase in pain symptoms.^[20–25]^ Patients with CM and AMO may have higher headache-related disability and rates of depression and anxiety; thus, it is crucial to treat and reduce the burden of illness in patients with CM and AMO.^[10]^ Excessive use of acute medications can also lead to the development of medication overuse headache (MOH).^[26]^

## 3. Screening

Fewer than 50% of all migraine patients are properly diagnosed, and underdiagnosis leads to unmet treatment needs.^[27]^ Approximately 53% of all visits for migraine occur in a primary care setting; thus, the primary care practice plays a crucial role in the appropriate diagnosis, treatment, and management of patients with migraine.^[27]^ A structured intake form is critical for this purpose and should include such attributes as sociodemographic characteristics, past medical history, family headache history, headache profile, and a basic diagnostic headache diary capturing headache frequency, severity, duration, features, and relevant precipitating factors.^[28,29]^ In the primary care setting, questionnaires, including the 3-item ID-Migraine screener and the 5-item Migraine Screen Questionnaire, can be used to assess the symptoms of migraine and aid diagnosis (Table 1).^[30,31]^

**Table 1:** Migraine screening tools and burden questionnaires.^[24,26–30]^

Migraine screening tools	Description
3-item ID-Migraine screener	•Assesses ○Headache disability ○Nausea ○Photophobia •A validated and reliable screening tool used in a primary care setting that may improve the recognition and rapid diagnosis of migraine
5-item ID-Migraine screener	•Assesses ○Frequency ○Characteristics of headache ○Migraine-related symptoms •Possible score 0-5 ○≥4 → indication of possible migraine
Migraine Disability Assessment questionnaire	•5-item questionnaire that assesses impact of migraine over the last 3 months on: ○Work/school attendance or productivity ○Household work performance or productivity ○Family/social/leisure activity attendance •Based on scores on these 5 items, disability is rated from Grade I (little/no disability; 0–5) to Grade IV (severe disability; ≥21)
6-item Headache Impact Test	•6-item questionnaire that assesses the impact of headache on: ○Severe headache frequency ○Daily activity limitations ○Fatigue, tiredness, irritability, and difficulty concentrating •Based on the scores for these 6 items, impact is classified as little to none (36–49) to severe impact (60–78)
Hospital Anxiety and Depression Scale	•14-item questionnaire that screens for the presence of anxiety or depression •A HADS score of ≥10 indicates depressive disorders and ≥13 indicates anxiety disorders
Beck Depression Inventory (BDI)	•21-item questionnaire that assesses the severity of depression •Based on BDI total scores, depression severity is rated from minimal (0–9) to severe (≥29)
9-item Patient Health Questionnaire	•9-item questionnaire screens for the presence of depression and severity of depressive symptoms •Based on total scores, severity of depressive symptoms ranges from minimal depression (1–4) to severe depression (≥20)
Migraine-Specific Quality of Life Questionnaire Version 2.1	•14-item questionnaire evaluates limitations in: ○Social activities ○Work-related activities ○Emotional well-being •Scores range from 0 to 100, with higher scores indicating better health-related quality of life

There are a number of additional validated questionnaires that can be used to complement these migraine screening tools to evaluate different aspects of disease burden for patients, including the severity of migraine- and headache-associated disability^[28]^ and the effects on quality of life (Table 1).^[32,33]^ Given the broad impact of migraine across all aspects of patients’ lives, particularly in patients with migraine complicated by the presence of psychiatric comorbidities or acute medication overuse, the regular clinical assessment of disability and quality-of-life outcomes is crucial. In addition, validated questionnaires are available to assess for the presence of psychiatric comorbidities, including the Beck Depression Inventory, 9-Item Patient Health Questionnaire (PHQ-9), and Hospital Anxiety and Depression Scale, in patients with migraine.^[34]^ The PHQ-9 and Hospital Anxiety and Depression Scale questionnaires are sensitive and specific for identifying comorbid depression in migraine and can be quickly and easily administered in clinical settings,^[34]^ allowing for appropriate management of depression in patients with migraine.

After symptom assessment and diagnosis of migraine, an appropriate treatment regimen should be established based on existing guidelines. the treatment regimen is based on the International Headache Society treatment guidelines.^[3]^

## 4. Preventive treatment options for migraine

Improving diagnosis and optimizing migraine treatment can significantly reduce disease burden.^[35]^ According to the American Migraine Prevalence and Prevention Study, patients with ≥6 headache d/mo, ≥4 headache d/mo with some impairment, or ≥3 headache days with severe impairment or requiring bedrest should receive preventive treatment for migraine.^[36]^ A number of medication classes used for preventive treatment, including antihypertensives, anticonvulsants, antidepressants, calcium channel blockers, and onabotulinumtoxinA, are not specific to migraine. The Quality Standards Subcommittee of the American Academy of Neurology and the American Headache Society evaluated the efficacy of medications from each of these classes based on published data.^[26]^ Only 3 antiepileptics (divalproex sodium, valproic acid, and topiramate), 3 beta-blockers (metoprolol, propranolol, and timolol), onabotulinumtoxinA, and frovatriptan (for the short-term prevention of menstrual migraine) are considered to have established efficacy based on data from ≥2 Class I studies.^[26]^ Propranolol, timolol, divalproex sodium, valproic acid, and topiramate are all approved by the US Food and Drug Administration (FDA) for the preventive treatment of EM,^[26]^ while onabotulinumtoxinA is FDA-approved for the preventive treatment of CM.^[26,37]^ These treatment options are not indicated primarily for migraine and are often limited by low persistence with treatment due to poor tolerability and lack of efficacy.^[38,39]^ Advances in the understanding of migraine pathophysiology have led to development of the first migraine-specific preventive therapies.^[35]^

### 4.1. Migraine-specific preventive treatment: CGRP pathway-targeted monoclonal antibodies (mAbs)

Monoclonal antibodies that target the CGRP pathway are disease-specific and mechanism-based migraine preventive treatments that have been shown to be safe and effective in patients with EM and CM.^[40–45]^ Four CGRP pathway–targeted mAbs have been FDA approved for the preventive treatment of migraine,^[40–43]^ including 3 targeting the CGRP ligand (fremanezumab, galcanezumab, and eptinezumab) and 1 targeting the CGRP receptor (erenumab).^[46]^ CGRP pathway–targeted mAbs are administered by subcutaneous injection (erenumab, fremanezumab, and galcanezumab) or intravenous infusion (eptinezumab) on a monthly (erenumab and galcanezumab) or quarterly (eptinezumab) basis; fremanezumab may be administered on a monthly or quarterly basis.^[40–43]^

**Figure 1.**
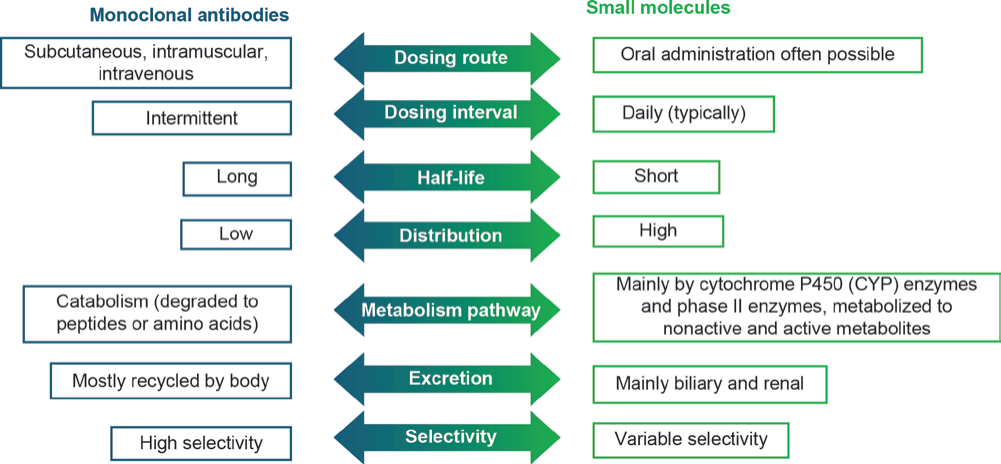
Pharmacological properties of mAbs and small molecules.^[48,49]^ mAbs = monoclonal antibodies.

CGRP pathway–targeted mAbs act on the CGRP pathway leading to reductions in headache pain and associated symptoms, while offering favorable tolerability.^[44,45,47]^ For clinical use, CGRP pathway–targeted mAbs differ from small molecule therapeutics in a number of ways, including higher target selectivity, which results in less off-target binding (Fig. 1). They also have a longer half-life (erenumab, 28 days; fremanezumab, 31 days; galcanezumab, 27 days; eptinezumab, 27 days), allowing for monthly or quarterly dosing, and are not metabolized by liver enzymes, which may reduce the potential for drug-to-drug interactions.^[50]^

### 4.2. Erenumab

**Figure 2.**
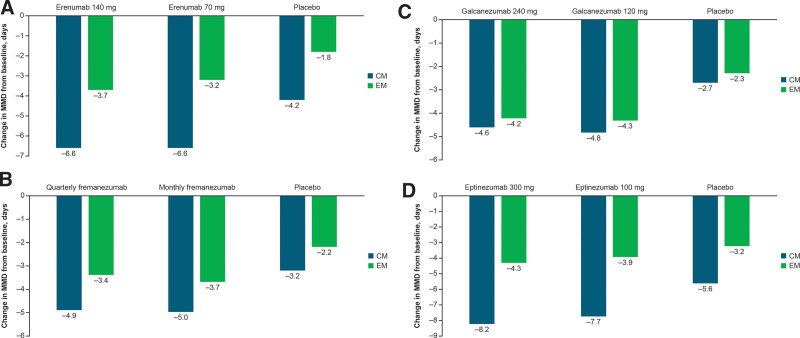
Change in MMD from baseline after 3 to 6 months of treatment with (A) erenumab^[51,52],a^; (B) fremanezumab^[53,54],b,c^; (C) galcanezumab^[55,56],d^; (D) eptinezumab.^[57,58],b^ CM = chronic migraine, EM = episodic migraine, MMD = monthly migraine days. ^a^Change from baseline to Months 2 to 3 for CM and from baseline to Months 4 to 6 for EM. ^b^Change from baseline to Month 3 for CM and EM. ^c^Quarterly fremanezumab, 675 mg; monthly fremanezumab, 225 mg. ^d^Change from baseline to Month 3 for CM and from baseline to Month 6 for EM.

Erenumab, which is administered as a monthly subcutaneous injection, is a human mAb (IgG2) targeting the CGRP receptor and was the first CGRP pathway–targeted mAb to be FDA approved for migraine prevention in adults.^[42]^ Erenumab was demonstrated to be safe and effective for the preventive treatment of CM and EM in randomized, placebo-controlled, phase 2 (CM) and 3 (EM: STRIVE and ARISE) trials, with significantly greater reductions in mean monthly migraine days (MMD) from baseline over 3 to 6 months with erenumab 70 mg or 140 mg versus placebo (all *P* < .001; Fig. 2A).^[51,52,59]^ Efficacy observed over 3 to 6 months in the pivotal studies was maintained over 1 year of treatment in a long-term extension study.^[60]^ The efficacy and tolerability of erenumab over 3 months have also been demonstrated in EM patients who have failed 2 to 4 prior prophylactic migraine treatments in the randomized, double-blind, placebo-controlled phase 3b LIBERTY study, with a mean MMD reduction of −1.8 with erenumab 140 mg versus −0.2 with placebo (*P* = .004).^[61]^

Erenumab has also demonstrated efficacy in patients with AMO or comorbid depression or anxiety. In a subgroup analysis of the phase 2 study in patients with CM (n = 667), erenumab treatment in patients with AMO (n = 274; 41%) resulted in significantly greater mean reductions from baseline in MMD and days with acute migraine-specific medication use than placebo at month 3 (*P* < .001; Table 2), as well as improvements in patient-reported outcomes.^[62]^ In the placebo, erenumab 70 mg, and erenumab 140 mg groups, respectively, the proportions of patients who reverted from AMO to no AMO at month 3 were 52%, 60%, and 71% for patients overusing simple analgesics; 33%, 65%, and 54% for those overusing triptans; and 40%, 45%, and 59% for those overusing combination therapy.

**Table 2:** Change in MMD from baseline in patients with AMO and comorbid depression and/or anxiety.

Migraine patients with AMO												
	Erenumab (CM)^[62]^			Fremanezumab (CM)^[63]^			Galcanezumab (CM/EM)^[64]^			Eptinezumab (CM)^[65],^*		
	Placebo	70 mg	140 mg	Placebo	Quarterly 675 mg	Monthly 225 mg	Placebo	120 mg	240 mg	Placebo	100 mg	300 mg
	(n = 117)	(n = 79)	(n = 78)	(n = 188)	(n = 201)	(n = 198)	(n = 353/173)	(n = 178/77)	(n = 177/84)	(n = 145)	(n = 139)	(n = 147)
MMD												
Mean change from baseline	–3.5	–6.6	–6.6	–2.8	–4.8	–5.2	–2.3/–2.7	–4.8/–6.3	–4.5/–5.8	–5.4	–8.4	–8.6
* P* value	—	<.001	<.001	—	.0002	<.0001	—	<.001/<.001	<.001/<.001	—	<.0001	<.0001
Days with acute medication use												
Mean change from baseline	–2.1	–5.4	–4.9	–3.1	–4.9	–5.5	–2.5/–2.7	–5.3/–6.3	–4.7/–5.9	—	—	—
* P* value	—	<.001	<.001	—	.0006	<.0001	—	.229/<.001	.301/<.001	—	—	—
**Migraine patients with comorbid depression and/or anxiety**												
	**Erenumab (CM**)**^[66]^**			**Fremanezumab (CM**)**^[67]^**			**Galcanezumab (CM**)**^[68]^**			**Eptinezumab**		
	**Placebo**	**70 mg**	**140 mg**	**Placebo**	**Quarterly 675 mg**	**Monthly 225 mg**	**Placebo**	**120 mg**	**240 mg**	**Placebo**	**100 mg**	**300 mg**
	**(n = 74**)	**(n = 58**)	**(n = 61**)	**(n = 67**)	**(n = 78**)	**(n = 96**)	**(n = 233**)	**(n = 127**)	**(n = 101**)	—	—	—
MMD												
Mean change from baseline	–1.3	–4.2	–4.1	–2.4	–5.4	–5.5	—	–2.1†	–1.9†	—	—	—
* P* value	—	<.001	<.001	—	<.001	<.001	—	<.001	<.001	—	—	—

AMO = acute medication overuse, CM = chronic migraine, EM = episodic migraine, MMD = average monthly migraine days, MOH = medication overuse headache.

* Patients with a comorbid MOH diagnosis.

† Least-squares mean difference versus placebo.

A post hoc analysis of patients with EM or CM and AMO enrolled in phase 2/3 clinical trials has also demonstrated significant reductions in medication overuse following erenumab therapy.^[63]^ In patients with EM who used acute headache medication at baseline, the reductions in mean monthly headache medication days over study months 4 to 6 were 1.5, 2.5, and 3.0 days in those receiving placebo, erenumab 70 mg, and erenumab 140 mg, respectively (*P* < .001 for erenumab vs placebo).^[63]^ Similarly, reductions in mean monthly headache medication days at month 3 among patients with CM who were acute medication users at baseline were 3.4, 5.5, and 6.5 days with placebo, erenumab 70 mg, and erenumab 140 mg, respectively (*P* < .001 for erenumab vs placebo).^[63]^ In both EM and CM patients with AMO at baseline, reductions in medication use with erenumab were particularly associated with reductions in use of migraine-specific medications.^[63]^ In a separate study by Tepper and colleagues, erenumab was shown to be effective in EM patients with depression and/or anxiety, with significantly greater reductions in MMD from baseline in the erenumab 70 and 140 mg groups compared with placebo (*P* < .001; Table 2).^[62,64]^

Real-world studies have confirmed the efficacy of erenumab for the preventive treatment of CM in patients with MOH.^[65–68]^ In a prospective multicenter study of 396 patients with CM and MOH receiving erenumab, a ≥50% reduction in monthly headache days (MHD) was achieved by 51% of participants and a ≥75% reduction in MHD was achieved by 20% of participants at 3 months following initiation of treatment.^[65]^ Erenumab therapy was also associated with significant decreases in mean monthly pain medication intake, disability (assessed using the 6-item Headache Impact Test), and mean pain intensity.^[65]^ Overall, 64% of participants reverted from CM with MOH to EM with no medication overuse at 3 months.^[65]^ In a retrospective, single-center study, erenumab therapy was associated with significant reductions in mean number of MHD and mean monthly analgesic use at 3, 6, 9, and 12 months following initiation of treatment with erenumab.^[66]^ Treatment was also associated with significant improvements in indices of quality of life and work-related difficulties.^[66]^

### 4.3. Fremanezumab

Fremanezumab, a humanized mAb (IgG2) targeting CGRP, has been FDA approved for the prevention of migraine in adults.^[40]^ Fremanezumab was demonstrated to be safe and effective for the preventive treatment of CM and EM in 2 pivotal randomized, placebo-controlled, phase 3 trials (HALO CM and HALO EM, respectively), with significantly greater reductions in mean MMD from baseline over 3 months with quarterly and monthly fremanezumab versus placebo (all *P* < .001; Fig. 2B).^[53,54]^ Reductions in MMD observed in CM and EM patients were maintained over 1 year of treatment in a long-term extension study.^[69]^ Fremanezumab was also shown to be effective in CM and EM patients with inadequate response to 2 to 4 prior preventive medication classes in the randomized, double-blind, placebo-controlled, phase 3b FOCUS study, with reductions in mean MMD of −3.7 with quarterly fremanezumab and 4.1 with monthly fremanezumab versus −0.6 with placebo (both *P* < .0001) over 3 months of double-blind treatment.^[70]^

Efficacy of fremanezumab was also evaluated in a subgroup of CM patients with AMO (n = 587; 52%) from the HALO CM study. In that subgroup, quarterly and monthly fremanezumab treatment resulted in significant reductions from baseline in mean MMD and days with acute medication use during 12 weeks of treatment compared with placebo (*P* ≤ .0006; Table 2).^[71]^ A significantly greater proportion of patients reverted to no AMO with fremanezumab treatment (quarterly, 55%; monthly, 61%) versus placebo (46%; *P* = .0389 and *P* = .0024, respectively).^[71]^

In a separate subgroup analysis of CM patients with comorbid moderate-to-severe depression (based on a PHQ-9 score ≥ 10; n = 241 [22%]) from the HALO CM study, fremanezumab treatment was associated with significantly greater reductions from baseline in mean MMD versus placebo (both *P* < .001; Table 2) over 12 weeks of treatment. For that subgroup, patients receiving fremanezumab experienced numerically greater reductions in depressive symptoms (based on mean PHQ-9 scores) than those receiving placebo, although these differences did not reach statistical significance (quarterly fremanezumab, –10.9 [77% reduction]; monthly fremanezumab, –9.8 [66%]; placebo,–9.2 [61%]; *P* = .113 and *P* = .558, respectively).^[72]^

Real-world studies have confirmed the effectiveness of fremanezumab for migraine preventive treatment in patients with AMO, depression, and anxiety.^[73,74]^ A US-based retrospective, panel-based online chart review included 220 patients with AMO, 134 patients with major depression disorder, and 120 patients with generalized anxiety disorder.^[73]^ In that study, reductions from baseline in mean MMD were observed as early as 1 month, with increasing reductions through 6 months in patients with AMO (mean [percent reduction] at 6 months: −10.1 [68.7%]), major depressive disorder (−9.9 [68.3%]), and generalized anxiety disorder (−9.5 [66.4%]). The ≥50% responder rates at 6 months were 78.8%, 81.8%, and 80.0% in the AMO, major depressive disorder, and generalized anxiety disorder subgroups, respectively.^[73]^ Further, in the major depressive disorder and generalized anxiety disorder subgroups, the severity of depression and anxiety improved based on patient report for 45.5% and 45.8% of patients, respectively, after starting fremanezumab treatment.^[73]^ In a separate retrospective study, among patients with comorbid depression and anxiety, significant reductions in the proportion of patients prescribed antidepressants and anxiolytics (both *P < *.05) were observed after fremanezumab initiation.^[74]^

### 4.4. Galcanezumab

Galcanezumab, a humanized mAb (IgG4) targeting CGRP was the third CGRP pathway–targeted mAb to be FDA approved for the preventive treatment of migraine in adults^[41]^ and has demonstrated efficacy and tolerability in randomized, double-blind, placebo-controlled, phase 3 studies in CM patients (REGAIN) and EM patients (EVOLVE-1 and -2).^[55,56,75]^ Galcanezumab treatment resulted in significant reductions in mean MMD in CM patients compared to placebo (*P* < .001; Fig. 2C).^[56]^ Treatment with galcanezumab also significantly reduced mean MMD for patients with EM compared to placebo (*P* < .001; Fig. 2C).^[75]^ The efficacy of galcanezumab has also been demonstrated in patients with episodic or chronic migraine and prior failure of medications from 2 to 4 different categories of preventive medications in the randomized, double-blind, placebo-controlled phase 3b CONQUER study, with reductions in mean MHD of −4.1 with galcanezumab compared with −1.0 with placebo (*P* < .0001) over the first 3 months of treatment.^[76]^

A subgroup analysis evaluated the safety and efficacy of galcanezumab in CM and EM patients with baseline AMO across the REGAIN (n = 708) and EVOLVE (n = 334) studies.^[77]^ Galcanezumab demonstrated significant reductions in MMD from baseline to month 3 in both CM and EM patients with AMO (all *P* < .001; Table 2).^[77]^ Among CM patients with AMO, medication overuse decreased by 48% for the 120 mg and 240 mg galcanezumab doses compared to 30% for placebo over 3 months.^[77]^ Among EM patients with AMO treated with 120 and 240 mg of galcanezumab, the proportion with AMO was reduced by 85% and 81%, respectively, compared to 64% with placebo over 6 months.^[77]^ In the CONQUER study in patients with prior failure of medications from 2 to 4 preventive medication categories, 46.6% and 43.0% of patients in the galcanezumab and placebo groups, respectively, had AMO at baseline, while 13.9% and 40.9% of patients, respectively, had AMO during the double-blind treatment period (*P* < .0001).^[78]^

In a subgroup analysis in EM patients with comorbid depression and/or anxiety, galcanezumab treatment resulted in significantly greater reductions in MMD from baseline compared with placebo (*P* < .001; Table 2).^[79]^ Reductions in MMD were also significantly greater with galcanezumab 240 mg than with placebo (*P* = .018; Table 2) in CM patients with depression/anxiety.^[79]^

### 4.5. Eptinezumab

Eptinezumab, a humanized mAb (IgG1) targeting CGRP that is administered intravenously on a quarterly basis, was the fourth FDA-approved CGRP pathway–targeted mAb for the prevention of migraine in adults.^[43]^ Two placebo-controlled phase 3 clinical trials of eptinezumab demonstrated efficacy for the preventive treatment of migraine (PROMISE-1 and -2).^[57,58]^ Treatment with eptinezumab 100 and 300 mg significantly reduced MMD in EM patients from baseline to month 3 compared to placebo (*P* = .00182 and *P* = .0001, respectively; Fig. 2D).^[57]^ A follow-up analysis showed that these reductions were maintained over 12 months of treatment.^[80]^ Similarly, in CM patients, both dosing regimens significantly reduced MMD compared to placebo, respectively (*P* < .0001; Fig. 2D).^[58]^ The efficacy of eptinezumab has also been demonstrated in patients with migraine and 2 to 4 prior migraine preventive treatment failures in the double-blind, placebo-controlled, phase 3b DELIVER study, in which significantly greater reductions in mean MMD were observed with eptinezumab (100 mg, −4.8; 300 mg, −5.3) compared with placebo (−2.1; both *P < *.0001) over 3 months.^[81]^

In a subgroup analysis of the PROMISE-2 study in patients with CM and a comorbid diagnosis of MOH, eptinezumab treatment resulted in significant reductions in MMD from baseline compared to placebo (both *P* < .0001; Table 2).^[82]^ Over the full 24-week treatment period, 51% of patients in the eptinezumab 100 mg group, 50% in the eptinezumab 300 mg group, and 27% in the placebo group reverted to no medication overuse.^[82]^ A post hoc analysis of the PROMISE-2 study also showed reductions in acute headache medication use in patients with CM and MOH receiving eptinezumab.^[83]^ Total monthly acute headache medication use was reduced by 49% in both eptinezumab arms (baseline, 21 d/mo; weeks 13–24, 11 d/mo) compared to a reduction of 29% in the placebo arm (baseline, 19.8 d/mo; weeks 13–24, 14.0 d/mo).^[83]^ Reductions were also seen in the use of triptans and simple and combination analgesics, with many patients reducing acute medication use to below the threshold for defining MOH.^[83]^ In this subgroup of patients with CM and MOH from the PROMISE-2 study, these improvements in migraine symptoms and reductions in acute medication use were accompanied by improvements in patient-reported outcomes, including an assessment of disability (6-item Headache Impact Test) and the patient global impression of change.^[84]^

### 4.6. Safety and tolerability of CGRP-pathway targeting mAbs

Overall, CGRP pathway–targeted mAbs are generally safe and well tolerated. In clinical trials, the incidence of adverse events (AEs) leading to discontinuation was <5% across all treatment groups for all CGRP pathway–targeted mAbs (Table 3). The most common AEs were injection-site reactions, upper respiratory tract infections, and nasopharyngitis (Table 3).^[52–59]^

**Table 3:** Summary of AEs (safety analysis set).

	CGRP pathway mAbs															
Erenumab^[44,45]^				Fremanezumab^[50,51]^				Galcanezumab^[52,59]^				Eptinezumab^[60,61]^			
CM^[45]^		EM^[44]^		CM^[51]^		EM^[50]^		CM^[52]^		EM^[59]^		CM^[61]^		EM^[60]^	
70 mg (n = 190)	140 mg (n = 188)	70 mg (n = 314)	140 mg (n = 319)	QLY (n = 376)	MLY (n = 379)	QLY (n = 291)	MLY (n = 290)	120 mg (n = 273)	240 mg (n = 282)	120 mg (n = 226)	240 mg (n = 228)	100 mg (n = 356)	300 mg (n = 350)	100 mg (n = 223)	300 mg (n = 224)
Patients with AEs, n (%)*																
≥1 AEs	83 (44)	88 (47)	180 (57)	177 (56)	265 (70)	270 (71)	193 (66)	192 (66)	159 (58)	160 (57)	147 (65)	163 (72)	155 (44)	182 (52)	141 (63)	129 (58)
Any SAEs	6 (3)	2 (1)	8 (3)	6 (2)	3 (<1)	5 (1)	3 (1)	3 (1)	1 (<1)	5 (2)	5 (2)	7 (3)	7 (1)		25 (3)	
AEs leading to treatment discontinuation	0	2 (1)	7 (2)	7 (2)	5 (1)	7 (2)	5 (2)	5 (2)	1 (<1)	4 (1)	5 (2)	9 (4)	3 (<1%)	8 (2)	6 (3)	5 (2)
Common AEs (occurring in ≥15% of patients in any group), n (%)																
Injection-site induration	—	—	—	—	74 (20)	90 (24)	57 (20)	71 (25)	—	—	—	—	—	—	—	—
Injection-site pain	7 (4)	7 (4)	10 (3.2)	1 (<1)	114 (30)	99 (26)	86 (30)	87 (30)	17 (6)	20 (7)	21 (9)	20 (9)	—	—	—	—
Injection-site erythema	—	—	—	—	80 (21)	75 (20)	55 (19)	52 (18)	4 (1)	13 (5)	6 (3)	7 (3)	—	—	—	—
Upper respiratory tract infection	5 (3)	6 (3)	21 (7)	15 (5)	18 (5)	16 (4)	11 (4)	16 (6)	9 (3)	9 (3)	13 (6)	12 (5)	15 (4)	19 (5)	22 (10)	23 (10)
Nasopharyngitis	6 (3)	3 (2)	31 (10)	35 (11)	19 (5)	15 (4)	11 (4)	11 (4)	17 (6)	9 (3)	19 (8)	16 (7)	19 (5)	33 (9)	17 (8)	14 (6)

AE = adverse event, CGRP = calcitonin gene-related peptide, CM = chronic migraine, EM = episodic migraine, mAb = monoclonal antibody, MLY = monthly, QLY = quarterly, SAE = serious adverse event.

* AEs were self-reported by patients in the erenumab, galcanezumab, and eptinezumab studies. In the fremanezumab studies, patients were proactively assessed for AEs.

In a retrospective, real-world analysis using data from the FDA Adverse Events Reporting System, the frequency of serious outcomes across CGRP pathway–targeted mAbs that were approved at the time the analysis occurred (i.e., erenumab, fremanezumab, and galcanezumab) was low (≤2%), and cardiovascular AEs were infrequent, with all individual cardiovascular AEs reported at a rate of ≤0.33 per 1000 exposed patients.^[85]^ The most commonly reported AEs included migraine/headache, drug ineffective, or injection-site reactions. While constipation was not among the top 10 AEs reported for fremanezumab or galcanezumab, it ranked second for erenumab and has been listed as a potentially serious AE in the prescribing information for erenumab.^[42]^ The prescribing information for all 4 CGRP pathway–targeted mAbs includes a precaution around the potential for severe hypersensitivity reactions; most reported hypersensitivity reactions were not serious, but some required discontinuation of treatment.^[40–43]^ Further, although CGRP pathway–targeted mAbs generally have a favorable cardiovascular safety profile,^[86–88]^ the prescribing information for erenumab includes a warning about the potential for new or worsening hypertension.^[42]^

## 5. Treatment of migraine with AMO or psychiatric comorbidities: discussion

CGRP pathway–targeted mAbs have demonstrated efficacy for preventive treatment in patients with migraine complicated by AMO^[62,63,71,77,82,83]^ or the presence of comorbid depression or anxiety^[64,72,79]^ in subgroup analyses of randomized, controlled clinical trials. As such, these studies were subject to certain limitations, including that they were post hoc analyses not prespecified in the corresponding study protocols; thus, the results of these studies should be interpreted with caution, given that they were not designed or powered to evaluate statistically significant differences between subgroups. Nevertheless, results of these subgroup analyses were generally consistent with the observed effects in the overall study populations.^[71,72,77]^ Further, there is now limited evidence from real-world studies in patients with AMO, MOH, and psychiatric comorbidities that support the findings of these subgroup analyses, demonstrating the effectiveness of CGRP pathway-targeted mAbs for preventive treatment of migraine in these populations.^[65–68,73,74]^

In the current review, we have restricted our discussion of treatment options for migraine complicated by AMO or the presence of psychiatric comorbidities to CGRP pathway–targeted mAbs, as the first disease-specific treatment options developed for the preventive treatment of migraine. Although additional small molecule CGRP pathway–targeted preventive treatments, atogepant and rimegepant, have been approved for the preventive treatment of migraine,^[89,90]^ there is currently a lack of published evidence for the efficacy of these treatments for patients with migraine with AMO or psychiatric comorbidities. Thus, these treatments were not included in this review.

It should be noted that there is a lack of direct comparative studies of the efficacy of CGRP pathway–targeted mAbs with other preventive treatments (e.g., antihypertensives, anticonvulsants, antidepressants, calcium channel blockers, onabotulinumtoxinA), including in patients with migraine with AMO or psychiatric comorbidities. In addition, the route of administration CGRP pathway–targeted mAbs may present a barrier for some patients due to injection anxiety, as noted for therapies in other disease states,^[91,92]^ and may also present a barrier in terms of access or costs. However, the lower frequency of dosing with these treatments compared with oral therapies may present a benefit in terms of medication burden and adherence or persistence.

## 6. Conclusions

Patients with migraine may face numerous hurdles when trying to manage their disease appropriately. This can be further challenged by the presence of psychiatric comorbidities (e.g., depression and anxiety) or AMO, often worsening symptom severity and disease burden. CGRP pathway mAbs, have been proven to be generally safe, well tolerated, and effective for migraine prevention across a broad range of patients, including those with disease complicated by the presence of psychiatric comorbidities and AMO. As a treatment class, CGRP pathway–targeted mAbs are well tolerated and offer benefits in terms of dosing frequency, requiring only monthly or quarterly dosing. Thus, these preventive treatments may be valuable options for reducing the overall burden of migraine in patients, including those who have had difficulty managing their disease, leading to overuse of acute medications, or those with depression or anxiety. Future real-world studies may shed additional light on the clinical use of CGRP pathway mAbs for patients with comorbidities or AMO who did not respond to an initial CGRP pathway–targeted mAb and need to switch between treatments within this class or for those who require concomitant treatment with another acute or preventive migraine medication.

## Acknowledgments

Editorial assistance was provided by Soan Kim, PharmD, of Cello Health Communications/MedErgy (Yardley, PA). This assistance was in accordance with Good Publication Practice (GPP3) guidelines and was funded by Teva Pharmaceuticals. The authors maintained full editorial control of the manuscript and the decision to submit it for publication.

## Author contributions

**Writing – review & editing:** Christopher Rhyne, Joshua M. Cohen, Michael J. Seminerio, Karen Carr, Lynda J. Krasenbaum.
